# Mutations in the NS5 RdRp Domain of Zika and Dengue Viruses: Insights into Molecular Patterns in Inland Midwestern Brazil

**DOI:** 10.3390/tropicalmed11030068

**Published:** 2026-03-03

**Authors:** José Henrique Francisco Roma, Rachel Cruz Alves, Bruno Moreira Carneiro, Renata Dezengrini Slhessarenko, Juliana Helena Chavez-Pavoni, Mariângela Ribeiro Resende

**Affiliations:** 1 Programa de Pós-graduação em Clínica Médica, Faculdade de Ciências Médicas, Universidade Estadual de Campinas, Campinas 13083-894, Brazil; mresende@fcm.unicamp.br; 2 Faculdade de Ciências da Saúde, Universidade Federal de Rondonópolis, Rondonopolis 78736-900, Brazil; rachel.alves@ufr.edu.br (R.C.A.); bruno@ufr.edu.br (B.M.C.); juliana.helena@ufr.edu.br (J.H.C.-P.); 3 Faculdade de Medicina, Universidade Federal de Mato Grosso, Cuiaba 78060-900, Brazil; renata.slhessarenko@ufmt.br

**Keywords:** arboviruses, phylogenetic analysis, molecular epidemiology, Zika virus, Dengue virus, genomic surveillance, mutations

## Abstract

In countries where Dengue virus is endemic, the occurrence of outbreaks and epidemic events is strongly associated with viral genomic evolution. In addition, the introduction of a new agent, such as Zika virus, in a naive population and its concomitant circulation may increase mutations and virulence. This study aimed to characterize the molecular patterns and circulation of Zika and Dengue viruses inland of midwestern Brazil. Samples from reported cases of zika and dengue fever were subjected to molecular and phylogenetic analyses. Partial genomes of these viruses were recovered and characterized from six samples. Phylogenetic analysis revealed that the Zika virus clustered within the American strain of Asian/American lineage and Dengue virus grouped within the Brazilian lineage (BR04) of serotype 2 from the Asian/American genotype. Amino acid substitutions, and consequently nonsynonymous mutations, were identified in the RdRp domain of the NS5 protein coding region in the recovered genomes from both viruses. These findings highlight the importance of molecular epidemiological surveillance, especially in endemic regions with cocirculation and substantial epidemic risk. Ongoing monitoring efforts are crucial to better understand viral evolution and its potential impact on future outbreaks and epidemic dynamics.

## 1. Introduction

Both *Orthoflavivirus zikaense* (ZIKV) and *Orthoflavivirus denguei* (DENV) are arthropod-borne viruses with a single-stranded positive-sense RNA genome comprising by a single open-reading frame (ORF) that encodes a polyprotein cleaved into three structural (C, prM and E) and seven nonstructural (NS1, NS2A, NS2B, NS3, NS4A, NS4B, NS5) proteins [1].

On the basis of differences in their amino acid sequences, ZIKV comprises two lineages, African and Asian/American, with the latter being subdivided into American, Pacific, and Southeast Asian strains. Regarding DENV, its classification includes four serotypes (DENV 1–4), with serotype 2 (DENV-2) characterized by six genotypes: American, Cosmopolitan, Asian/American, Asian I, Asian II, and Sylvatic. In addition to this classification, the Asian/American genotype of DENV-2 is represented by four lineages: BR01-BR04 [1,2,3].

During recent decades, as an important neglected tropical disease, DENV has been responsible for extensive outbreaks and epidemics, with a significant increase in severe and fatal cases especially following the introduction of serotype 2, which is strongly associated with more severe clinical manifestations [4,5,6].

Similarly, the introduction of ZIKV into Brazil represented a highly complex event, given the virus’s pronounced neurotropism, which facilitates infection of central nervous system cells and triggers inflammatory responses. This pathogenic mechanism can disrupt normal fetal brain development during pregnancy, leading to Congenital Zika Syndrome (CZS), and has also been associated with the onset of Guillain-Barré Syndrome (GBS) in affected individuals. Although there has been a significant reduction in the number of cases since 2017, this virus still circulates in the Americas and may pose a health risk during pregnancy [7,8,9,10].

Located in the central region of Brazil and serving as a strategic connection point to all parts of the country, the state of Mato Grosso (MT) plays a significant role in the dissemination of infectious diseases. However, molecular investigations of arboviruses in this region remain limited. Molecular studies are essential for understanding viral evolution and determining factors associated with virulence. In this study, we analyzed the genomes of Zika virus (ZIKV) and Dengue virus serotype 2 (DENV-2), which were obtained from reported arbovirus cases during the first ZIKV outbreak in the Americas in a municipality situated around the geographical coordinates of 16°28′15″ S and 54°38′08″ W in the southeastern MT state, according to the Brazilian Institute of Geography and Statistics (IBGE) [11].

## 2. Materials and Methods

### 2.1. Study Area and Sampling

Rondonópolis, a city located in southeastern of Mato Grosso state, in the midwestern of Brazil, belongs to tropical weather Amazon region and has been experiencing successive arbovirus outbreaks.

In this study, serum samples were collected during an epidemiological surveillance action between 2015 and 2016, a period corresponding to the introduction of ZIKV in Brazil. A Total of 179 samples from patients who presented with clinical symptoms and suspected arbovirus infection were included, which were reported according to the Brazilian Notifiable Diseases Information System of the Ministry of Health of Brazil (MoH) [12]. Samples were collected by convenience up to the fifth day after the onset of symptoms and stored at −80 °C in the Laboratório de Virologia Clínica e Aplicada of Universidade Federal de Rondonópolis (UFR) until molecular investigation. Samples with insufficient volume for molecular analysis and inadequate storage conditions were excluded.

Epidemiological data of reported ZIKV and DENV cases in Mato Grosso and Rondonópolis were obtained from the SINAN database [12]. The research was approved by the Research Ethics Committee (protocol number 10767319.8.0000.8088/26 April 2019) of the Universidade Federal de Mato Grosso (UFMT).

### 2.2. Laboratory Analysis

An aliquot of 150 µL of each serum sample was subjected to ribonucleic acid (RNA) purification via silica column protocol (NucleoSpin^®^ RNA Virus, Macherrey-Nagel, Duren, Germany) according to the manufacturer’s instructions.

Reverse transcription (RT) with a final volume of 20 µL was carried out from 10 µL of purified RNA as template, via random primers (Invitrogen^®^, Waltham, MA, USA), and the mixture was incubated at 25 °C for 5 min, followed by incubation at 42 °C for 60 min. Complementary deoxyribonucleic acid (cDNA) was stored at −20 °C until use.

Nested RT-PCR and a multiplex nested RT-PCR, which target the NS5 coding region, were performed for the detection of ZIKV and DENV, respectively, via specific sets of primers previously designed [13,14,15]. The results were previously confirmed by real-time assay (RT-qPCR) via the ZDC Multiplex RT-PCR Assay^®^ (Bio-Rad, Hercules, CA, USA) according to the manufacturer’s instructions.

An electrophoresis with a two percent agarose gel stained with ethidium bromide was accomplished, and the amplicon size was compared against a 100 bp DNA ladder (Promega^®^, Madison, WI, USA) under ultraviolet light. The amplicon products were purified via NucleoSpin^®^ Gel and PCR Clean-up (Macherrey-Nagel, Duren, Germany) according to the manufacturer’s instructions.

### 2.3. Genome Sequencing

Genome sequencing was carried out from aliquots of amplified cDNA products by RT-PCR, using forward and reverse primers by Sanger method with a 3500 Genetic Analyzer^®^ Applied Biosystems (Waltham, MA, USA). Initially, the quality of the generated sequences of each sample was inspected and trimmed through a chromatogram via MEGA software (version 12.0) against the strains of the ZIKV (NC_035889) and DENV (NC_001474) reference genomes, which were available in the GenBank database. The consensus of the six sequences were subsequently edited and deposited in GenBank under the accession numbers of PQ219523, PQ219524, and PQ219525 for ZIKV and PQ186549, PQ186550, and PQ186551 for DENV.

### 2.4. Phylogenetic Analysis

For phylogenetic analysis, the nucleotide sequences were queried against the NCBI database via BLAST software (version 2.17.0). Complete and partial sequences that covered each lineage and strain of ZIKV and Brazilian lineages and genotypes of DENV-2 were included in separate phylogenetic analyses. The retrieved sequences, in addition to the reference genomes of ZIKV and DENV, were trimmed for analysis only in the open reading frame (ORF) region.

Genomes from other described lineages and strains of ZIKV, and Brazilian clades within Asian/American and the other genotypes of DENV were included in the analysis. In addition, sequences of the ZIKV African genotype and *Spondweni virus*, and the serotypes I, III and IV of DENV, were used as outgroups to characterize the retrieved genomes of ZIKV and DENV-2, respectively.

Multiple sequence alignment was carried out via the online server MAFFT (version 7), and the analysis of nucleotide variation, amino acid changes, and coding position was performed. A maximum likelihood (ML) phylogenetic tree was subsequently constructed with 1000 bootstraps via MEGA (version 12.0) and FigTree software (version 1.4.4) for figure generation.

### 2.5. Spatial Analysis

A Kernel density map was created considering the location of notifications in the neighborhood’s household of patients through the municipality of Rondonópolis. The map was constructed via open-source QGIS software (version 3.42.2).

## 3. Results

**Figure 1:**
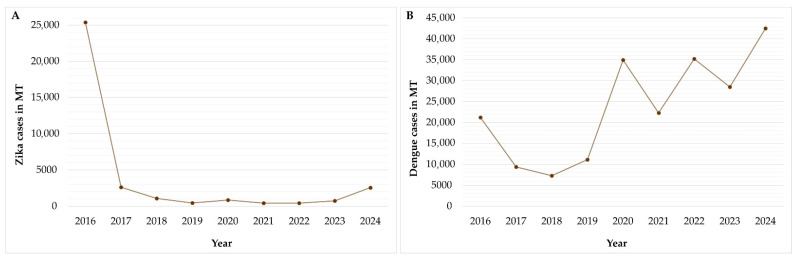
Annual series of zika fever (**A**) and dengue fever (**B**) reported cases in Mato Grosso state since the introduction of ZIKV in Brazil.

In Brazil, approximately 265,000 probable cases of zika fever were recorded between 2015–2016. During the same period, the state of Mato Grosso (MT) reported high incidence rates of zika and dengue fever, with 750 and 896 cases per 100,000 inhabitants, respectively. As expected, most notifications occurred during the rainy season in the early months of 2016. However, the incidence of zika fever has markedly decreased in recent years, with 12.3 cases per 100,000 inhabitants reported in 2024. In contrast, dengue fever remains highly prevalent, reaching an incidence of 1119.9 cases per 100,000 inhabitants, as illustrated in Figure 1 [12,16].

In our study, a total of 179 samples were collected between August 2015 and August 2016 in the southeastern region of Mato Grosso state and subjected to a previous molecular evaluation [15]. From six positive samples, partial genomes were successfully recovered (10.7%), of which three were identified as DENV and three as ZIKV, and underwent to phylogenetic analysis.

Among the six recovered genomes, most patients (5 out of 6) were female, with ages ranging from 23–45 years. The nucleotide sequences exhibited approximately 97.4% to 97.7% similarity compared with the ZIKV (NC_035889) and DENV (NC_001474) reference genomes available in the GenBank database (Table 1).

**Figure 2:**
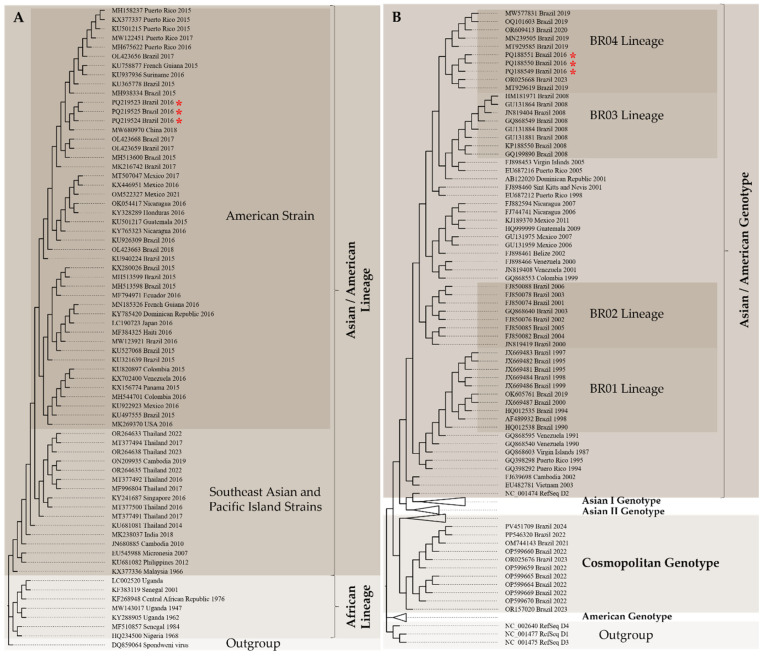
Maximum likelihood phylogenetic trees of ZIKV (**A**) and DENV (**B**) based on the recovered genomes in this study (highlighted by asterisks).

A maximum likelihood (ML) phylogenetic analysis was performed on the six partially recovered genomes. The ML tree for ZIKV revealed that the strains circulating in southeastern Mato Grosso clustered within the American strain from the Asian/American lineage. Similarly, the ML tree constructed from DENV genomes clustered within the BR04 lineage of the serotype 2 from the Asian/American genotype, alongside other genomes previously reported from the North, Northeast, Southeast, and Midwest regions of Brazil (Figure 2).

**Figure 3:**
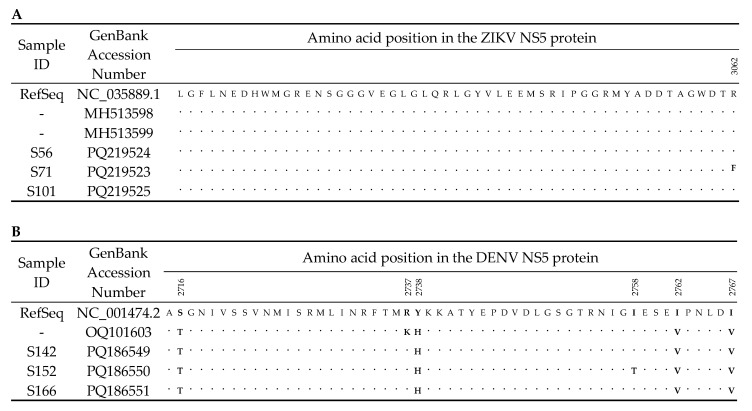
Amino Acid nonsynonymous mutations in the partial NS5 protein of ZIKV (**A**), and DENV (**B**) from this study compared with NCBI RefSeq sequences and sequence accession numbers OQ101603; MH513598; MH513599. Dots and letters (bold) indicate amino acid identities and mismatches, respectively. The amino acid positions were determined from the ORFs of the RefSeq sequences.

Amino acid changes were identified in all the recovered genomes, indicating the presence of nonsynonymous mutations. In the ZIKV genomes, one amino acid substitution (R3062F) was detected in the NS5 coding region, despite variations at seven nucleotide positions. In contrast, analysis of the DENV NS5 coding region revealed five amino acid substitutions (S2716T, Y2738H, I2758T, I2762V, and I2767V) compared with the reference genome available in GenBank (NC_001474). Additionally, one amino acid change (R2737K) observed in the DENV genome reported in at least one sequence from Mato Grosso during the 2019 Brazilian outbreak (GenBank accession OQ101603) was still conserved in our samples in 2016 compared with the RefSeq genome (NC_001474) [17]. These DENV amino acid substitutions resulted from a total of 38 nucleotide changes across the analyzed genomes (Figure 3).

**Figure 4:**
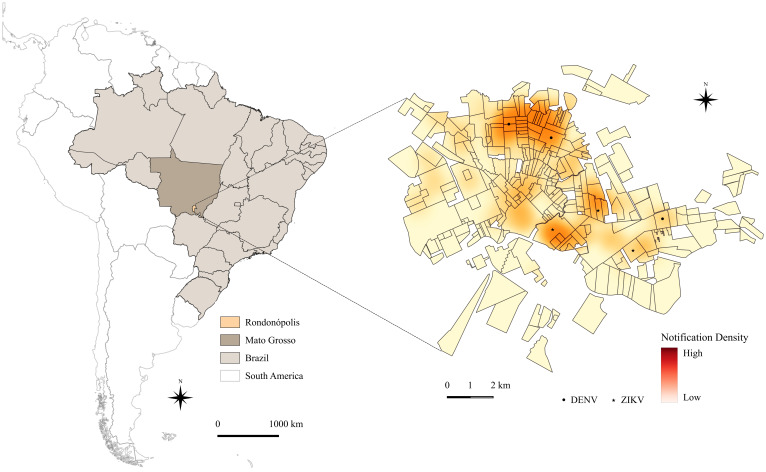
Kernel density map of reported ZIKV and DENV cases across neighborhoods in the municipality of Rondonópolis during the 2015–2016 period. Maps of Brazil, the state of Mato Grosso and Rondonópolis city (orange) located in the midwestern region of the country, with the recovered nucleotide sequences of ZIKV (★) and DENV (●) highlighted.

In the spatial analysis, the Kernel density map illustrated a high concentration of notifications in the most populous neighborhoods across the municipality. The samples sequenced in this study are highlighted on the map. The recovered ZIKV genomes were associated with cases from the central and northeastern regions of the city, whereas DENV genomes were obtained from cases in the northern and northeast neighborhoods, as shown in Figure 4.

## 4. Discussion

Since the introduction of ZIKV in the Americas, phylogenetic analyses have been widely performed to elucidate its molecular epidemiology, particularly in the context of the cocirculation of DENV and other arboviruses. Moreover, the emergence of ZIKV in Brazil was characterized by an unprecedented increase in cases of Congenital Zika Syndrome and other associated clinical manifestations that had not been previously recognized [7,8].

Although a considerable number of cases were documented during the initial introduction of ZIKV, Brazil has subsequently experienced a marked decline in its incidence, whereas DENV has continued to circulate extensively, maintaining a high prevalence [12,16].

Several factors may be related to this scenario, such as the probable occurrence of competition between these viruses and other arboviruses, such as Chikungunya virus (CHIKV) and DENV, because they share the same vector in endemic areas, as suggested by Fuller et al. [18] in Rio de Janeiro. In addition, the immunity acquired after the ZIKV introduction in Brazil could be another condition associated with the decrease in reported cases, and even with misdiagnosis based on clinical–epidemiological data. These finding are clearly reflected in the NCBI database, without further inclusion of new viral genome sequences from notified cases in Brazil from 2019 [16].

In this context, importantly, the occurrence of the cosmopolitan genotype of DENV-2 in Brazil was first reported in 2021. Its introduction likely occurred in 2020 from the northern region of the country, along with its subsequent dissemination across all regions of the Brazilian territory in the following years (Figure 1B) [18,19,20].

Furthermore, it is important to emphasize the need for molecular laboratory diagnosis to accurately distinguish between different arboviral agents, as these viruses often cause similar clinical symptoms. Relying solely on clinical, epidemiological, and serological approaches may lead to inaccurate diagnoses, although such methods have been widely used for reporting suspected cases in Brazil [15,21].

Regarding the distribution of reported cases in the municipality of Rondonópolis in 2016, although there is a well-established association between socioeconomic vulnerability and high rates of infectious diseases, often related to the unplanned and disorderly growth of urban areas, a high incidence of notifications was also observed in the central region of the municipality. this area is characterized by a relatively high socioeconomic level, intense population movement may favor viral circulation, given the urban transmission dynamics of these arboviruses [22,23,24].

The introduction of a virus into a naive population can lead to a rapid and widespread epidemic due to the lack of preexisting immunity, which has serious consequences for health, as observed with the entrance of ZIKV in 2015–16 in Brazil [8,25,26]. In addition, the endemicity of a virus is conditioned by an evolutionary process driving the generation of important genetic mutations, as both ZIKV and DENV serotype 2 (DENV-2) have circulated in Brazil since 2015 and 1990, respectively [27,28].

For both ZIKV and DENV, the encoder region of NS5 is the largest nonstructural protein and has important functions, such as RNA methyltransferase, RNA-dependent RNA polymerase (RdRp), and genome replication activities. Furthermore, RdRp plays an important role in generating genetic variability in RNA viruses, such as ZIKV and DENV [1,3].

Although the NS5 is considered the most conversed region in the genome, since their first description, ZIKV and DENV have shown nucleotide changes and consequently amino acid mutations, implicating on its virulence and pathogenicity [27]. In this sense, although many mutations have been reported in the ZIKV genome, to the best of our knowledge, the influence of the nonsynonymous mutation described in our study, which is located in the RdRp domain, remains unknown [29,30].

Furthermore, the changes found in the ZIKV genome from our samples were not reported in other sequences from samples collected in Mato Grosso. This observation could be related to the geographic origin of the strain detected in our study, since the other sequences reported in the MT likely originated in the states of Paraíba and Tocantins, as proposed by Vieira et al. [31], whereas the phylogeographic reconstruction by Costa et al. [32] also indicates the states of Pernambuco, São Paulo, and Pará as possible routes of ZIKV entry into the MT. This further reinforces the importance the geographic location of the MT and the numerous possible entry routes of viral agents contributing to its molecular diversity and evolution.

With respected to DENV, of the five nonsynonymous mutations found in our samples, which were partially located in both domains of the methyltransferase component of the capping enzyme and RdRp, four (S2716T; Y2738H; I2762V; I2767V) were still conserved in samples collected throughout Brazil, as well as Mato Grosso state during the 2019 outbreak [16]. Furthermore, we detected an amino acid mutation (I2758T) apparently not previously described in the literature and therefore not considered characteristic of the BR04 lineage. Instead, it appears to be unique to the sample analyzed in this study. Although the impact of these mutations on DENV virulence has not yet been reported, they may reflect ongoing evolutionary dynamics, as they are located within the methyltransferase and RdRp domains of the viral genome, which play critical roles in replication efficiency and, consequently, in overall viral fitness [1,16,33].

In addition, even though the three recovered DENV sequences appear to establish a distinct cluster, it is premature to conclude that they represent a novel cluster within the BR04 lineage. This interpretation should be approached with caution, given that the analysis is based on a relatively short genomic fragment, which constrains the strength of the phylogenetic inferences.

The main limitation of this study is related to partial genome recovery. Importantly, the samples analyzed in this study were collected between 2015 and 2016 during a public surveillance epidemiological program in reason of the introduction of ZIKV in the Americas and its particular importance to public health. However, the molecular analysis of this study was conducted only between 2019 and 2020, which might have contributed to molecular degradation. Therefore, assays to obtain larger fragments were not successful, and only shorter sequences were recovered and used for phylogenetic analysis. Similarly, low ZIKV viral loads, as evidenced by high Ct values observed via qPCR, represent a technical limitation for Sanger sequencing. In addition, it is important to mention the small sample size as a limitation in the inference of evolutionary patterns.

## 5. Conclusions

This study aimed to investigate the genetic diversity of ZIKV and DENV in an inland region of Brazil. As expected, the viral sequences analyzed corresponded to the BR04 lineage of the Asian/American DENV-2 genotype and the Asian/American lineage of ZIKV. Despite the relatively short genomic fragments obtained in our study compared with the complete genome of the *Orthoflavivirus* genus, we identified nonsynonymous mutations within a region typically regarded as highly conserved, which are fundamental to viral replication competence and, consequently, to overall viral fitness. Therefore, functional studies and long-term follow-up are essential to determine whether these specific mutations play a role in the immunogenicity or pathogenicity of both ZIKV and DENV.

These findings underscore the critical role of molecular epidemiological surveillance, particularly in endemic areas where ZIKV and DENV cocirculate and pose a significant epidemic threat. Continuous monitoring based on molecular surveillance, including sentinel sequencing through the sampling of a representative fraction of confirmed cases within the municipality, should be implemented in conjunction with the assessment of changes in epidemiological patterns and spatiotemporal analyses to detect the introduction or emergence of new lineages. Additionally, the incorporation of entomological indicators and strengthened vector control measures is important. Therefore, genomic–epidemiological integration is essential for elucidating viral molecular evolution and its implications for future outbreaks and epidemics.

## Figures and Tables

**Table 1:** Data from ZIKV and DENV positive samples analyzed from southeastern Mato Grosso state during the 2015–2016 ZIKV epidemic.

Sample ID/Virus	GenBank Accession Number	Ct Value	Collection Date	Days of Symptoms	Amplicon Size (bp)	Nucleotide Identity (%)	Age	Gender
S71/ZIKV	PQ219523	36.69	03/2016	2	161	97.45 *	35	F
S56/ZIKV	PQ219524	35.07	02/2016	3	156	97.44 *	30	F
S101/ZIKV	PQ219525	34.94	03/2016	2	156	97.44 *	23	F
S142/DENV	PQ186549	26.65	03/2016	5	320	97.77 **	32	M
S152/DENV	PQ186550	21.28	01/2016	4	321	97.45 **	45	F
S166/DENV	PQ186551	22.70	03/2016	4	317	97.77 **	29	F

* GenBank Accession Number: NC_035889. ** GenBank Accession Number: NC_001474.

## Data Availability

The original contributions presented in this study are included in the article. Further inquiries can be directed to the corresponding author.
